# Annual surveys for point-prevalence of healthcare-associated infection in a tertiary hospital in Beijing, China, 2012-2014

**DOI:** 10.1186/s12879-016-1504-4

**Published:** 2016-04-18

**Authors:** Yaowen Zhang, Jing Zhang, Dong Wei, Zhirong Yang, Yanyan Wang, Zhiyuan Yao

**Affiliations:** Infection Management and Disease Prevention Department, China-Japan Friendship Hospital, Beijing, 100029 China; Center of Post-marketing Safety Evaluation, Peking University Health Science Center, Beijing, 100083 China; Department of Epidemiology and Bio-statistics, Peking University Health Science Center, Beijing, 100083 China; Department of Medical Records and Statistics, China-Japan Friendship Hospital, Beijing, 100029 China; Infection Management and Disease Prevention Department, China-Japan Friendship Hospital, 2 East Yinghuayuan Street, Chaoyang District Beijing, 100029 China

**Keywords:** Healthcare-associated infection, Prevalence, China

## Abstract

**Background:**

This study aimed to investigate the prevalence of healthcare-associated infection (HAI) in the China-Japan Friendship Hospital, a tertiary level hospital in Beijing, China.

**Methods:**

We defined HAI using the criteria established by the Ministry of Health of the People’s Republic of China. Three cross-sectional surveys were conducted from 2012 to 2014. Inpatients who had been hospitalized for at least 48 h were surveyed. Information on HAI prevalence, isolated pathogens and use of antibiotics were collected. Logistic regression models were used to assess the associations between HAI and potential risk factors.

**Results:**

During three cross-sectional surveys, a total number of 4,029 patients were included (1,233 patients in 2012, 1,220 patients in 2013 and 1,576 patients in 2014). The overall prevalence of patients with HAI was 3.6 % (95 % confidence interval (CI) 3.1 %–4.2 %). Respiratory tract infections were the most common type (64.7 %) of HAIs, followed by urinary tract infections (12.6 %) and bloodstream infections (5.4 %). HAI occurrences were significantly associated with male sex (odds ratio (OR) = 2.25, 95 % CI 1.53-3.32), age over 85 years (OR = 4.74, 95 % CI 2.54–8.83), hospitalization in the intensive care units (ICUs) (OR = 2.42, 95 % CI 1.31–4.49), indwelling urinary catheter (OR = 4.21, 95 % CI 2.46–7.20) and mechanical ventilation (OR = 2.31, 95 % CI 1.30–4.09). Gram-negative bacteria were found to be the most isolated pathogens (67.1 %), with gram-positive bacteria and fungi accounted for 20.3 % and 10.5 %, respectively. Antibiotics were administered to 34.3 % of the included patients over the study period.

**Conclusions:**

The overall HAI prevalence in our hospital is similar to previous studies that were conducted in other areas of China, and the respiratory tract infection should be the priority in HAI reduction control within China. We should focus HAI reduction efforts on patients with advanced age, hospitalization in the ICU and indwelling devices.

## Background

Healthcare-associated infection (HAI) continues to be a major public health concern worldwide [1]. As a common cause of morbidity and mortality among hospitalized patients, HAI is associated with prolonged hospital stay and increased healthcare cost [2, 3]. Surveillance has been recognized as an important method to reduce HAI. A well-conducted prospective longitudinal surveillance combined with infection control program is an ideal approach to reduce the incidence of HAI and to reduce its associated costs accordingly [4–6]. However, it is expensive and time consuming to conduct prospective surveillance. In contrast, cross-sectional survey is relatively more cost-effective to obtain basic HAI information, such as prevalence, isolated pathogens and antibiotic uses [7]. In addition, in countries with limited resources, especially for hospitals that are not yet able to carry out surveillance on the incidence of HAI, cross-sectional studies are preferred to determine the magnitude of HAI and are necessary to initiate effective prevention and control program. Furthermore, repeated cross-sectional surveys could provide meaningful data to investigate potential trends of HAI [8, 9].

Currently, several cross-sectional surveys have been conducted in Anhui and Hubei Province in China [10–12]. However, there have been no published records so far on HAI in Beijing, the capital of China, which has a relatively large number of hospitals and patients [13]. In order to better understand HAI epidemiology and to improve further infection control program, we conducted three annual cross-sectional surveys on HAI in the China-Japan Friendship Hospital, a tertiary hospital in Beijing from 2012 to 2014.

## Methods

### Ethics statement

The protocol of our study was approved by the Ethical Review Committee of China-Japan Friendship Hospital. This observational study did not affect the clinical course of treatment, and all the data was analyzed anonymously. Thus, neither verbal nor written informed consent was obtained from participants. We were also exempt from requiring ethical approval due to delinking of personal identifiers from clinical and laboratory data.

### Study design

China-Japan Friendship Hospital, a tertiary hospital founded in 1984 in Beijing, China, is comprised of 43 clinical departments (13 medical departments, 12 surgical departments, 10 traditional Chinese medical departments, five Intensive care units, one pediatric department, one Gynecology/obstetrics department and one Ophthalmology/otorhinolaryngology department), with 1,377 hospital beds in 2012, 1,405 in 2013 and 1,747 in 2014. The hospital admitted 44,825 inpatients in 2012, 50,263 in 2013 and 59,308 in 2014. It also serves 2.3 million outpatients every year. Three single day cross-sectional surveys were conducted by the Infection Management and Disease Prevention Department on May 16^th^ 2012, Sep 2^nd^ 2013 and May 21^st^ 2014. With the same methodology, these three cross-sectional surveys adopted an identical questionnaire designed by Beijing Quality Control Center for the Management of Hospital Infection. Patients who had been admitted to the hospital for at least 48 h at 8 a.m. on the survey days were included.

### Case definition

HAI was defined according to the criteria established by the Ministry of Health (MOH) of the People’s Republic of China [14], and was a modification of the definition from the USA Centers for Disease Control and Prevention (CDC) [15]. According to the CDC’s definition, respiratory tract infection (RTI) contains cases of “pneumonia” and “lower respiratory tract infection other than pneumonia” only, whereas the MOH’s version also includes “Upper respiratory infection” if a patient has a fever with higher than 38 °C. Moreover, while patients below 12 months of age are considered as a separate group in the CDC’s definition, they are included in the overall sustainable population for HAI in the MOH’s definition, except for cardiovascular and central nervous system infections.

In our study, infection occurred after hospital admission (≥48 h) that was neither present nor incubating on admission was considered as HAI. The main outcomes were common HAIs, including RTI, urinary tract infection (UTI), bloodstream infection (BSI), surgical site infection (SSI), digestive tract infection (DTI), skin and soft tissue infection (SSTI) and “Others” (which could not be classified into any of above infections). Where the same patient had more than one type of infection, they were counted in each separate category.

Isolated microorganisms implicated in infections were categorized as colonizers or pathogens according to medical records and patients’ symptoms. Patients who were receiving antibiotics on the survey day were identified. In addition, antimicrobial treatment was recorded if the antimicrobial agent was still prescribed on the treatment chart at the time of survey. In the case of surgical prophylaxis, any single dose of an antimicrobial agent given within the 24-h period before 8:00 a.m. on the survey day was recorded. Antiviral drugs and tuberculostatics were beyond the scope of our research.

### Data collection

A team working in the Infection Management and Disease Prevention Department that consisted of a clinical microbiologist, an epidemiologist, two physicians, and two public health physicians, had overall accountability for the study. The team was responsible for the training and supervision of the infection control investigators in each ward. The director of the team was in charge of study design and data collection.

Before the survey, definitions of the investigated variables in the questionnaire were circulated to all involved investigators and all investigators agreed on the interpretation of the diagnostic criteria. The importance of data completeness was highlighted among those investigators who collected data.

Data was extracted from all relevant sources including clinical records, temperature charts, laboratory reports and radiographs. Additionally, investigators could request additional information from nurses, doctors, or pharmacists.

Information was filled in via the Hospital Information System. The questionnaire contained four sections, including 1) demographics, 2) presence of HAI and organisms responsible for HAI (when appropriate), 3) exposure to invasive devices (central/peripheral intravascular catheter, Indwelling urinary catheter and mechanical ventilation) and 4) antibiotic prescription. Exposure to invasive devices was defined as using the devices for at least 48 h at 8 a.m. on the survey days.

Surveys were checked after completion by the team to avoid missing data. A return visit was performed if patients or medical records were temporarily unavailable at the onset of investigation. Infection reports were followed up thereafter to investigate suspected microorganisms.

### Data analysis

Continuous variables are presented as mean ± standard deviation, and categorical variables are shown as percentages. The prevalence of HAI (defined as the number of infected patients divided by the total number of patients) and the constituent ratio of a specific HAI (defined as a specific type of HAI divided by the total number of HAI) were assessed. The 95 % confidence intervals (CI) for each parameter were also estimated.

For potential risk factors, variables with a *P*-value of less than 0.05 in a univariate analysis were selected and included in a multivariate logistic regression model. Odds ratio (OR) and 95 % CI were evaluated to assess associations between risk factors and HAI.

Statistical analyses were performed using SAS 9.13 (SAS Institute Inc., Cary, NC). All tests were two-tailed and *P*-value less than 0.05 was considered as statistically significant.

## Results

During the three point-prevalence surveys, 1,233 patients in 2012, 1,220 patients in 2013 and 1,576 patients in 2014, with a total number of 4,029 patients were included. Of these, 50.2 % were male. Ages of patients ranged from two days to 106 years. The mean age was 55.9 ± 19.2 years. Out of all participants, 1,457 were admitted to medical department, 1,199 were in surgical department, 892 were in traditional Chinese medical department, 120 were in ICUs, 73 were in the pediatric department, 65 were in the gynecology/obstetrics department, and 223 were in the ophthalmology/otorhinolaryngology department.

### Prevalence and infection sites

Overall, 147 of 4,029 patients were diagnosed as HAI. Among the 147 infected patients with a total of 167 infected sites, single infection occurred in 130 patients (88.4 %), double infections in 15 patients (10.2 %), triple and quadruple infections both in one patient (0.7 %). The overall prevalence of HAI was 3.6 % (95 % CI, 3.1 %–4.2 %) (Table 1).

**Table 1:** Prevalence of HAI in a tertiary hospital in Beijing, China

	2012 (*n* = 1,233)	2013 (*n* = 1,220)	2014 (*n* = 1,576)	Total (*n* = 4,029)
N [% (95 % CI)] of patients with HAI^a^	34 [2.8 (1.8–3.7)]	63 [5.2 (3.9–6.4)]	50 [3.2 (2.3–4.0)]	147 [3.6 (3.1–4.2)]
N [% (95 % CI)]of HAIs^b^	42 [3.4 (2.4–4.4)]	69 [5.7 (4.4–7.0)]	56 [3.6 (2.6–4.5)]	167 [4.1 (3.5–4.8)]
N (constituent ratio) of sites with HAI				
RTI	23 (54.8)	48 (69.6)	37 (66.1)	108 (64.7)
UTI	9 (21.4)	6 (8.7)	6 (10.7)	21 (12.6)
BSI	3 (7.1)	3 (4.3)	3 (5.4)	9 (5.4)
SSI	2 (4.8)	4 (5.8)	2 (3.6)	8 (4.8)
DTI	2 (4.8)	1 (1.4)	3 (5.4)	6 (3.6)
SSTI	0 (0.0)	3 (4.3)	2 (3.6)	5 (3.0)
Others	3 (7.1)	4 (5.8)	3 (5.4)	10 (6.0)

*HAI* healthcare-associated infection, *RTI* respiratory tract infection, *UTI* urinary tract infection, *BSI* bloodstream infection, *SSI* surgical site infection, *DTI* digestive tract infection, *SSTI* skin and soft tissue infection

^a^Number and proportion of infected patients in all patients

^b^Number and proportion of infected sites in all patients

RTI was the most common type of HAI (64.7 %), followed by UTI (12.6 %), BSI (5.4 %), SSI (4.8 %), DTI (3.6 %) and SSTI (3.0 %). Other infections accounted for 6.0 % (Table 1).

### Risk factors

Univariate analyses showed that HAI prevalence was significantly higher in patients of male sex (5.0 %), older than 85 years (18.7 %) and hospitalized in ICUs (35.0 %). Additionally, patients who were exposed to central/peripheral intravascular catheter, indwelling urinary catheter or mechanical ventilation also had a significantly higher prevalence of HAI (11.3 %, 12.1 %, 15.7 %, respectively) (Table 2).

**Table 2:** Univariate and multivariate analyses comparing patients with and without HAI in relation to potential risk factors

Characteristics (*N*)	With HAI (*n* = 147) (*N*, %)	Without HAI (*n* = 3,882) (*N*, %)	Crude OR (95 % CI), *P*	Adjusted OR (95 % CI), *P* ^a^
Sex				
Female (2,007)	45 (2.2)	1,962 (97.8)	1	1
Male (2,022)	102 (5.0)	1,920 (95.0)	2.32 (1.62–3.31), *P* < 0.05	2.25 (1.53–3.32), *P* < 0.05
Age				
≤ 17 years (133)	1 (0.8)	132 (99.2)	0.35 (0.05–2.58), *P* = 0.31	0.55 (0.07–4.55), *P* = 0.58
18–34 years (506)	6 (1.2)	500 (98.8)	0.56 (0.24–1.32), *P* = 0.19	0.82 (0.34–1.95), *P* = 0.65
35–64 years (1,949)	41 (2.1)	1,908 (97.9)	1	1
65–84 years (1,307)	99 (6.9)	1,342 (93.1)	2.79 (1.89–4.12), *P* < 0.05	2.19 (1.44–3.32), *P* < 0.05
≥ 85 years (134)	25 (18.7)	109 (81.3)	10.67 (6.26–18.20), *P* < 0.05	4.74 (2.54–8.83), *P* < 0.05
Wards				
Medicine (1,457)	47 (3.2)	1,410 (96.8)	1	1
Surgery (1,199)	26 (2.2)	1,173 (97.8)	0.67 (0.41–1.08), *P* = 0.10	0.29 (0.17–0.52), *P* < 0.05
Traditional Chinese Medicine (892)	32 (3.6)	860 (96.4)	1.12 (0.71–1.76), *P* = 0.64	1.27 (0.79–2.05), *P* = 0.32
ICU^b^ (120)	42 (35.0)	78 (65.0)	16.15 (10.05–25.96), *P* < 0.05	2.42 (1.31–4.49), *P* < 0.05
Pediatrics (73)	0 (0.0)	73 (100.0)	-	-
Gynecology/Obstetrics (65)	0 (0.0)	65 (100.0)	-	-
Ophthalmology/Otorhinolaryngology (223)	0 (0.0)	223 (100.0)	-	-
Central/Peripheral arteriovenous catheter				
No (3,532)	91 (2.6)	3,441 (97.4)	1	1
Yes (497)	56 (11.3)	441 (88.7)	4.80 (3.39–6.80), *P* < 0.05	1.43 (0.87–2.36), *P* = 0.16
Indwelling urinary catheter				
No (3,402)	71 (2.1)	3,331 (97.9)	1	1
Yes (627)	76 (12.1)	551 (87.9)	6.47 (4.63–9.06), *P* < 0.05	4.21 (2.46–7.20), *P* < 0.05
Mechanical ventilation				
No (3,710)	97 (2.6)	3,613 (97.4)	1	1
Yes (319)	50 (15.7)	269 (84.3)	6.92 (4.82–9.95), *P* < 0.05	2.31 (1.30–4.09), *P* < 0.05

*HAI* healthcare-associated infection, *ICU* intensive care unit, *OR* odds ratio

^a^Adjusted for all included variables

^b^Refers to General ICU, Cardiovascular ICU, Emergency ICU, Kidney ICU and Respiratory ICU

Multivariate logistic regression analyses indicated male sex, age of older than 85 years, hospitalization in ICUs, indwelling urinary catheter and mechanical ventilation were significantly associated with HAI. Crude OR and adjusted OR with 95 % CI were listed in Table 2.

### Isolated pathogens

Some of HAI episodes were diagnosed according to physical symptoms and/or radiographs, and 66 % (97/147) of infected patients were found the presence of pathogens in culture. In total, 143 pathogenic bacteria types were isolated. Gram-negative bacteria were found to be the most common isolated pathogens (67.1 %, 96/143), followed by gram-positive bacteria, fungi, and others (such as viruses) accounted for 20.3 % (29/143), 10.5 % (15/143) and 2.1 % (3/143) of the pathogens, respectively. *Pseudomonas aeruginosa* accounted for 18.9 % (27/143) of isolates and was the most common cause of HAI, followed by *Acinetobacter baumannii* (17.5 %, 25/143) and *Klebsiella pneumoniae* (9.8 %, 14/143) (Table 3).

**Table 3:** Distribution of HAI pathogens in a tertiary hospital in Beijing, China, 2012–2014

Pathogens	N of pathogens, by infection sites							
	RTI	UTI	BSI	SSI	DTI	SSTI	Others	Total
Gram-negative bacteria	72	10	4	2	3	1	4	96
*Pseudomonas aeruginosa*	25	2	0	0	0	0	0	27
*Acinetobacter baumannii*	22	0	0	1	1	0	1	25
*Klebsiella pneunoniae*	11	1	1	0	0	0	1	14
*Escherichia coli*	1	5	2	1	0	0	1	10
*Stenotrophomonas maltophilia*	6	0	0	0	1	0	0	7
Others	7	2	1	0	1	1	1	13
Gram-positive bacteria	15	2	3	2	2	3	2	29
*Staphylococcus aureus*	4	0	0	2	0	2	1	9
CNS	3	1	0	0	1	0	1	6
*Enterococcus spp.*	2	1	1	0	0	0	0	4
*Streptococcus spp.*	3	0	0	0	0	0	0	3
Others	3	0	2	0	1	1	0	7
Fungus	11	3	0	0	0	1	0	15
*Candida albicans*	7	1	0	0	0	0	0	8
Others	4	2	0	0	0	1	0	7
Others	3	0	0	0	0	0	0	3
Total	101	15	7	4	5	5	5	143

*HAI* healthcare-associated infection, *CNS* Coagulase-negative *Staphylococcus*, *RTI* respiratory tract infection, *UTI* urinary tract infection, *BSI* bloodstream infection, *SSI* surgical site infection, *DTI* digestive tract infection, *SSTI* skin and soft tissue infection

### Antibiotic use

Antimicrobials were administered in 34.3 % (1,380/4,029) of patients over the study period. Among patients who received antimicrobials, 48.2 % (665/1,380) were used for prophylaxis, 41.7 % (576/1,380) were used for treatment, and 10.1 % (139/1,380) were used for both purposes (Table 4).

**Table 4:** Antibiotic use in a tertiary hospital in Beijing, China

	2012 (*n* = 1,233)	2013 (*n* = 1,220)	2014 (*n* = 1,576)	Total (*n* = 4,029)
Antibiotics use (*N*, %)	445 (36.1)	414 (33.9)	521 (33.1)	1,380 (34.3)
Purpose of antibiotic use, (*N*, %)				
Prophylactic	226 (50.8)	182 (44.0)	257 (49.3)	665 (48.2)
Treatment	179 (40.2)	199 (48.1)	198 (38.0)	576 (41.7)
Treatment & Prophylactic	40 (9.0)	33 (8.0)	66 (12.7)	139 (10.1)

## Discussion

HAI is recognized as a public health threat, as well as a safety issue for hospitalized patients nationally and internationally. This was the first attempt to review the epidemiology of HAI in a tertiary level hospital in Beijing, with the same standardized methodology. Our study demonstrated that the overall prevalence of patients with HAI was 3.6 %. Our results were relatively stable in three annual surveys, which provides basic information to further evaluate the infection control program in our hospital. Although variety in methodologies and study population between different studies and comparable infection rates require adjustment for intrinsic and extrinsic risk factors, the HAI prevalence in our hospital was similar to studies conducted in other areas of China, in which the MOH’s definition was used [10–12]. Our results were also similar to that of conducted in the USA [16], but lower than other studies carried out in Italy, Greek, Malaysia, England, Wales, Northern Ireland and the Republic of Ireland (from 6.1 % to 13.9 %), in which the CDC’s definition was used [17–20]. Differences between the prevalence data reported by other countries and our survey may be explained by the differing lengths of hospital stay, definitions of HAIs, sizes of hospitals, and methods for measuring HAI.

Consistent with previous studies with similar design in China [10–12], RTI was found to be the most common HAI. In those studies conducted in China, including ours, the HAI definition established by MOH was used. The MOH’s definition, modified based on definition from CDC [15], is similar to the CDC’s definition but with some alterations. In the MOH’s definition, RTI contains cases of “pneumonia”, “lower respiratory tract infection other than pneumonia”, as well as “upper respiratory infection” if a patient has a fever with higher than 38 °C. The broader RTI definition used by the MOH is likely to result in a higher prevalence of RTI in western countries, compared to the definition adopted by the CDC. The data of our study was comparable to other countries’ results except for RTI.

Similar with previous investigations [21–23], our study showed that patients older than 85 years of age, hospitalized in ICUs, indwelling urinary catheter and mechanical ventilation were significantly associated with an increased risk of HAI. Therefore, strategies of infection control in our hospital should be focused on these factors in the future. Male sex as a risk factor in our analysis was an interesting finding, which was also found to be significant in Balkhy’s study [23]. However, this finding was inconsistent with some other studies [21, 22]. This result needs to be confirmed by other researchers. Length of stay, a significant potential risk factor for HAI, was not collected in our study because we thought in a cross-sectional study, it would be hard to explain whether a longer hospital stay would generate an infection, or an infection would result in a longer hospital stay. It’s noteworthy that, some other important risk factors associated with HAI were not considered in the questionnaire, such as underlying disease, duration of invasive devices, conscious state, glucocorticoid use, and immunoglobulin use, and these need further exploration.

Microbiological documentation was available for 66 % of infected patients. This proportion is higher than previous results [10, 11, 21]. Overestimating the prevalence of HAI might be possible, as some of HAI episodes were diagnosed by physical symptoms and/or radiographs. Gram-negative bacteria seemed more responsible than Gram-positive bacteria for isolated HAI. It might be due to the abuse of antimicrobials both before and during the hospital admission. According to China Food and Drug Administration’s report, about 30 % of people use antimicrobials at home to treat a common cold or diarrhea [24]. Use of antimicrobials in hospital is also high, with the proportion of 54.8 % in 2001 and 46.6 % in 2010 [25]. The proportion of meticillin-resistant *Staphylococcus aureus* (MRSA) among all *Staphylococcus aureus* was 44.4 %, but there were only 9 isolates of *Staphylococcus aureus* in total and it is therefore hard to draw a clear conclusion. In contrast to MRSA, the major isolated pathogen within infected patients in Greek, Morocco, and Saudi Arabia was *Pseudomonas aeruginosa* [18, 21, 26]*,* In addition, *Acinetobacter baumannii, Escherichia coli* and *Klebsiella pneunoniae* were also common in China [10–12].

In a point-prevalence survey of antibacterial use in 20 European hospitals, 30.1 % of inpatients were receiving antibacterial on the survey day [27]. Another point prevalence survey revealed that 34.6 % were receiving at least one antimicrobial agent [28]. Consistent with above studies, our study found 34.3 % of patients were receiving antimicrobials at the time of survey. Previously, abuse of antimicrobial was a major concern in China [24, 25]. Currently lower proportion of antimicrobial use may be due to the new policy about antibiotic management, initiated by the MOH in May 2011 aiming to standardize antibiotic consumption and to reduce antibiotic overuse [29]. The stringent policies includes restricting clinical indications on antibiotic use and defining daily dose (DDD) under 40 per 100 patients per day, limiting antimicrobial varieties in hospitals, and cutting off economic connections between pharmaceutical companies and doctors.

However, the proportion of patients who receive antibiotic prophylaxis is still very high since antibiotic prophylaxis was associated with a decreased risk of SSI, which may result in higher mortality and morbidity. Many surgeons tend to extend the duration of prophylactic antibiotic use, despite the clinical guidelines recommend discontinuation of prophylactic antibiotics within 24 h after surgery. SSI rates after surgery have been observed to range from 30 % to 60 % without prophylactic antibiotics, but prophylactic antibiotic use could dramatically reduce the risk of SSI [30–32]. So it might be that a higher antibiotic prophylaxis use rate lead to a lower SSI rate in our hospital.

Several limitations of our study should be noted. Firstly, patients with longer duration of hospital stay were more likely to have their infection details recorded, compared to patients with shorter stay. Thus, it is possible that the persistent infections were overestimated while the temporary infections were underestimated. Secondly, cross-sectional study does not allow to conclude causality between different factors and HAI. Last, it is impossible to calculate the HAI incidence rate with a cross-sectional design.

## Conclusions

Despite the limitations mentioned above, our study yielded valuable data on the epidemiology of HAI, including point-prevalence, potential risk factors, isolated pathogens and antibiotic use among inpatients in a tertiary hospital in Beijing, China. The overall HAI prevalence in our hospital is similar to previous studies that were conducted in other areas of China, and the respiratory tract infection should be the priority in HAI reduction control within China. We should focus HAI reduction efforts on patients with advanced age, hospitalization in the ICU and indwelling devices.

## Abbreviations

95 % CI: 95 % confidence intervals
BSI: bloodstream infection
CDC: centers for disease control and prevention
CNS: coagulase-negative *Staphylococcus*
DDD: defining daily dose
DTI: digestive tract infection
HAI: healthcare-associated infection
ICU: intensive care unit
MOH: ministry of health of the People’s Republic of China
MRSA: meticillin-resistant *Staphylococcus aureus*
OR: odds ratio
RTI: respiratory tract infection
SSI: surgical site infection
SSTI: skin and soft tissue infection
UTI: urinary tract infection
